# One Health in Hainan, China: Urgent need and current progress

**DOI:** 10.1016/j.onehlt.2024.100688

**Published:** 2024-01-26

**Authors:** Jiaolin Bao, Yuxi Tian, Ren-Bo Ding

**Affiliations:** a One Health Institute, Collaborative Innovation Center of One Health, Hainan University, Haikou 570228, China; b Key Laboratory of Tropical Biological Resources of Ministry of Education, School of Pharmaceutical Sciences, Hainan University, Haikou, 570228, China

## 1 Emerging infectious diseases are major global health threats

Human development has been accompanied by the prevalence of various infectious diseases for centuries. In the last thirty years, emerging infectious diseases have increasingly become a major threat to global public health security, about 75% of them are zoonotic diseases that transmitted from animals to humans [1]. Several novel emerging infectious diseases, such as SARS (2002), swine flu-H1N1 (2009), Ebola (2014), MERS (2015), ZIKV (2016), COVID-19 (2019), MPXV (2022), Hendra, Nipah and Marburg, have triggered global pandemics and caused millions of deaths. Since December 2019 that the global COVID-19 pandemic started, COVID-19 has affected more than 220 countries, infected about 771 million people and caused over 6.97 million deaths [2], which posed irreversible and significant damages to global economies, politics, societies and cultures. Although over three years have passed, the COVID-19 pandemic still continues to disrupt people's daily life, and China is one of the worst affected countries during this pandemic.

## 2 One Health concept for solving global public health problems

One Health is a collaborative, multisectoral, and transdisciplinary approach working at the local, regional, national, and global levels, with the goal of achieving optimal health outcomes recognizing the interconnection between people, animals, plants, and their shared environment [3]. As the process of globalization accelerates and international exchanges become more frequent, the relationship between humans, animals and the environment has also become closer, and human beings are facing the serious challenge of many public health problems together [4]. In recent years, complex public health problems such as emerging infectious diseases, population explosion, regional development imbalance, land resource abuse, antibiotic resistance and abnormal climate require more actions of public health management with global response. In this context, One Health has emerged as a new approach in response to the global public health crises, and it is a new strategy for multi-institutional, interdisciplinary, and cross-regional collaboration and communication that innovatively addresses public health issues from a holistic perspective of biological and environmental health, so as to better respond to public health problems that cannot be effectively solved by a single discipline, a single department or a single region, and to safeguard the balance and unity of human health, animal health and environmental health.

## 3 The urgent need to build One Health system in Hainan, China

China is a high-risk area for emerging infectious diseases. During the past 20 years, China has experienced major outbreaks of SARS, highly pathogenic avian influenza (i.e. H5N1 and H7N9), and COVID-19. Many other important zoonotic pathogens are also present in China, especially in southern China, such as Hainan, which may cause potential new outbreaks in the future due to the combination of high population density, hot climate, intensive livestock production, abundant wildlife, land-use changes and habitat fragmentation. As one of the world's largest consumers of antibiotics, China also carries a high risk of antibiotic resistance [5]. Emerging infectious diseases are becoming a major threat to China's development and public safety, so there is an urgent need for Hainan to serve as a pilot region for the whole country and the world to build a standardized proactive epidemic prevention and control system in One Health approach.

Hainan is the China's largest free trade port, an important fulcrum of the “One Belt, One Road” global strategy, and is being built as the window and new benchmark for China's all-round reform and opening up in the new era. In 2025, the Hainan Free Trade Port will launch island-wide independent customs operations and initiate all-round opening up, which gives full play to the role of a bridge between China and countries along the “One Belt, One Road” and countries of Association of Southeast Asian Nations in terms of economic, trade and cultural exchanges and cooperation. Meanwhile, Hainan is building a global transit base for animal and plant germplasm resources, which will become the main channel for new species and germplasm resources from all over the world to enter China [6]. This means that Hainan will be confronted with massive movements of people, animals, plants and goods, and will be more susceptible to emerging infectious diseases, zoonotic disease outbreaks and biosecurity threats. Hainan is an independent island located in the southernmost part of China, and is the only tropical monsoon climate province in China. The unique climatic condition, ecosystem and location of Hainan island provide a natural cradle for the emergence and spread of tropical diseases. As such, it also provides an extensive pool of pathogenic resources for research on zoonotic infectious diseases and insect-borne infectious diseases under One Health concept. In Hainan, it is more conducive to the traceability of pathogens and outbreak monitoring, and to the establishment of a globally influential pilot demonstration of One Health system.

## 4 Current progress on One Health in Hainan, China

### 4.1 Governmental policy support to build cross-department One Health system

In 2020, the Central Committee of the Communist Party of China and the State Council of China issued the “Overall Plan for the Construction of Hainan Free Trade Port”, which states that Hainan will be support: (a) to accelerate the construction of public health prevention, control and treatment systems; (b) to establish monitoring and early warning, emergency response platforms and decision-making command systems for infectious diseases and public health emergencies; (c) to improve early prevention, risk analysis and timely handling capabilities; (d) to establish a cooperation mechanism on joint prevent and control of disease outbreaks through collaboration among multiple departments [7]. In 2021, the People's Government of Hainan Province promulgated the “Outline of the Hainan Province 14th Five-Year Plan (2021-2025) for National Economic and Social Development and the Long-Range Objectives Through the Year 2035”, which explicitly proposes to establish a global pilot demonstration of One Health practice and build a stronger public health system. The specific measures include: (a) to form a holistic view of health that encompasses human, animal and environment by comprehensively introducing One Health concept; (b) to establish a globally influential standardized One health system in terms of institutional mechanism innovation, interdisciplinary joint research, talent cultivation and international cooperation in governance; (c) to build a platform for monitoring and early warning of infectious diseases and public health emergencies, as well as an emergency response platform and a decision-making command system [6]. Both central and local governments have provided a series of policy supports for Hainan to build One Health system with cross-department collaboration.

### 4.2 Increasing academic strength on reinforcing interdisciplinary One Health research

Faced with the new concept of One Health, in order to strengthen the capacity building of global public health governance, corporately address the public health security challenges, and build an interdisciplinary One Health system, several well-known universities have newly established One Health institutes with multi-disciplinary integration in Hainan, including Shanghai Jiao Tong University, University of Edinburgh, Hainan University, Hainan Medical University, and others. In May 2020, Shanghai Jiao Tong University, University of Edinburgh and the Hainan Boao Lecheng International Medical Tourism Pilot Zone jointly established the SJTU-UE International Joint Research Center of One Health. In February 2021, Hainan University together with the Hainan Provincial Center for Disease Control and Prevention, the Hainan Provincial Center for Animal Disease Prevention and Control and Haikou People's Hospital jointly established the One Health Institute of Hainan University. In June 2022, Hainan University further integrated One Health relevant collages and disciplines to establish the Collaborative Innovation Center of One Health. Hainan Medical University formed One Health Research Center in June 2021, restructured International School of Public health and One Health. In November 2021, Hainan Medical University launched the first academical journal in One Health field in China named “*One Health Bulletin*”. A series of efforts from multiple academic institutes have been done, which all aim to expand the interdisciplinary collaboration and communication on One Health research incorporating human, animal and environmental health.

### 4.3 The World Bank's One Health Pilot Demonstration Project

In 2021, the World Bank approved $300 million loan for China to combat emerging infectious diseases in One Health approach, with $175 million invested in Hainan Province [8]. The World Bank chose Hainan to implement this $175 million project because Hainan has accelerated institutional integration and innovation, demonstrated leadership in effective cross-department collaboration and is well positioned to implement this challenging concept. The One Health Hainan Pilot Demonstration Project (i.e. the World Bank Emerging Infectious Diseases Prevention, Preparedness and Response Project in Hainan Province) will become the largest real-world implementation project in China, create a global leading demonstration significance of the One Health governance system, reduce the risk of zoonotic and other emerging health threats, and promote Hainan's all-round deepening of reform and opening up.

## 5 Prospective

Hainan has a unique geographic location, a specific open-door policy and abundant talent resources, and obvious advantages in implementing One Health practice. However, there are still insufficiencies in the capacity of responding to public health threats for Hainan. There is an urgent need to take the opportunity of the One Health Hainan Pilot Demonstration Project to formulate and implement a series of new strategies and measures through cross-department and multi-disciplinary coordination and cooperation, as well as utilizing the legislative power of the Hainan Free Trade Port to accelerate the construction of the One Health law and regulation system. It is necessary to promote upgrading of the governance system and capacity in the field of One Health, to achieve cross-border, efficient, coordinated and information-based management for responding to infectious diseases and public health emergencies. It is worth looking forward to the future of One Health practice in Hainan, which not only promotes the economic and social development of Hainan Free Trade Port, but also provides the world with a One Health pilot demonstration with Chinese characteristics.

## Funding

This work was funded by 10.13039/501100001809National Natural Science Foundation of China (No. 82203206), Hainan Provincial Natural Science Foundation of China (No. 822QN300), Hainan University Collaborative Innovation Center Research Fund (No. XTCX2022JKB01), Hainan “Nanhai New Star” Project (No. 202309009) and 10.13039/501100005693Hainan University High-level Talent Start-up Fund (No. KYQD(ZR)-21169).

## Author's contributions

All authors contributed equally to the conceptualization, drafting, critical reading and revision of the manuscript, and gave final approval for publication.

## Declaration of competing interest

The authors declare no competing interests.

## Data Availability

No data was used for the research described in the article.
